# Summary of Remarks on Fractures of the Shaft of the Femur in Adults

**Published:** 1877-12

**Authors:** Frank H. Hamilton


ART. III.—*Summary of Remarks on Fractures of the shaft of the Femur in Adults.* Reported by JAMES COCHRANE LAY, M. D. M.R.C.S.E.

By invitation of Prof. Frank H. Hamilton, I attended his clinic at Bellevue Hospital, on Wednesday last, to hear what he had to say in regard to the treatment of the above mentioned fracture. The learned Doctor exhibited many splints and appliances which were in use in former times, to keep the fractured bones in apposition, spoke of the great benefit to humanity realized by the discovery of the method of making extension with adhesive plaster, commonly termed "Buck's extension," which at the present time, owing to modifications and improvements, may as justly be called by any other name, still originally used and described by Dr. Crosby of Hanover. After mentioning the fact which is well known to the profession, that fractures of the shaft of the femur in adults are almost universally, very oblique, thereby causing great difficulty in maintaining due apposition of the fractured extremities, the Doctor exhibited a half dozen or more cases now under treatment, causing them to be brought into the Theatre on their narrow beds, from his surgical wards. Speaking of counter extension, the Dr. remarked that all the plans hitherto adopted of crutches in the perineum, perineal bands, or Plaster of Paris bandages, none now seemed to him to be equal to the counter extension obtained by the weight of the body, on elevating the foot of the bed about four inches and causing the patient to lie flat on the bed with the pillow *under the head only.* The extension employed is effected by a weight of from 15 to 22 lbs. attached to a cord, which passes over a pulley, and from thence to a small piece of wood, about two inches wide, and long enough to prevent the extending adhesive strips from causing lateral pressure on the ankle. The limb is then bandaged from the toes to the knee, where the adhesive strips should terminate. The fractured bones are to be maintained in their natural position by means of four moulded light strips applied to the four aspects of the thigh, and held in position by small bands passing around the whole, and tied over the front splint. Then the Doctor exhibited one of his patients, with the apparatus above described, and also the long splint, which he calls the splint "par excellence" of all. It consists of a long straight piece of pine board about three-fourths of an inch thick, and about four and a half inches wide, the lower end of which is securely fastened to a cross piece of wood long enough to extend both sides of the splint nearly across the bed, say two and a half feet; this cross piece is used to prevent eversion of the foot and limb, which is so common an event. The long splint is well padded and secured by strips of bandage to the whole limb, and above to the trunk as high as the 6th or 7th ribs by broad bandages encircling the body, thereby keeping the limb in a straight line with the body, and the bones in a direct line. With this method of treatment the Doctor stated that he had no difficulty in securing the most favorable results. In closing his remarks the Doctor alluded, he said with gratification to the fact, that he had just received a copy of the fifth edition of his work on Fractures and Dislocations, translated into the German language and published at Gottingen, a compliment which he mentioned, in order to show the appreciation of those in the old country, for the efforts of those in the new.

